# Gas sensors and real-time video for accurate classroom occupancy detection

**DOI:** 10.1016/j.mex.2025.103386

**Published:** 2025-05-28

**Authors:** Koundinya Challa, Issa W. AlHmoud, Chandra Jaiswal, Balakrishna Gokaraju, Raymond Tesireo

**Affiliations:** North Carolina A&T State University, 1601 E Market St, Greensboro, North Carolina, 27411, 3363347, USA

**Keywords:** HVAC efficiency, Deep neural networks, Real-time occupancy detection, YOLOv4 object detection, Smart classroom, Computer vision, Energy conservation, Indoor environment monitoring, OpenCV integration, Multimodal sensor fusion, Multimodal Occupancy Detection and HVAC Optimization

## Abstract

This study introduces an advanced methodology for optimizing HVAC efficiency through real-time classroom occupancy detection by combining video analysis with gas sensor data to enhance accuracy and reliability. The proposed system integrates video feeds captured by a Logitech C20 webcam with data from an MS1100 gas sensor module, ensuring a dual-modal approach to occupancy detection. A YOLOv4 object detection model, trained on a diverse dataset of over 20,000 labeled human face images, achieves over 98 % accuracy in identifying and counting occupants in real time. OpenCV is employed to facilitate efficient and seamless processing of video streams, enabling the system to deliver real-time results crucial for dynamic HVAC control. The integration of gas sensor data addresses scenarios where environmental factors, such as low light or obstructions, could impair video analysis, thereby improving detection reliability under diverse conditions. The combination of these modalities provides a robust and adaptable framework for occupancy detection, which can be scaled for different building types and configurations. This method demonstrates significant potential in reducing energy consumption and enhancing the sustainability of building management systems by providing precise occupancy data for HVAC optimization. The approach offers a practical and scalable solution for the growing demand for energy-efficient infrastructure in smart buildings. The architecture ensures seamless integration between visual and environmental sensing modalities, enhancing real-time responsiveness and occupancy detection reliability.

• Utilizes a YOLOv4 object detection model and MS1100 gas sensor for real-time occupancy detection.
• Achieves over 98 % accuracy with a dataset of over 20,000 labeled human face images.
• Offers a scalable and efficient solution for energy-efficient HVAC systems.

## Graphical abstract

**Figure:**
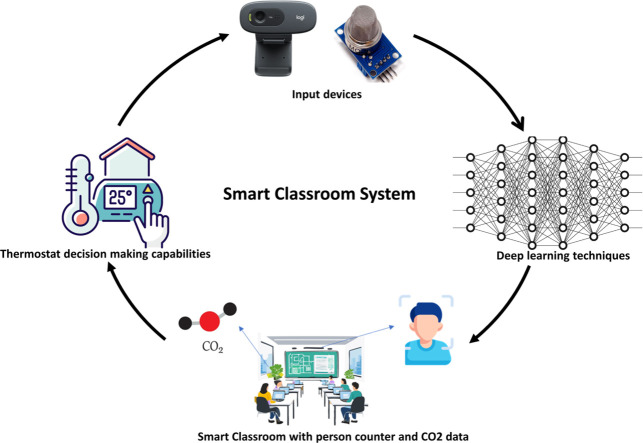

Specifications table

**Table:** 

Subject area:	Energy
More specific subject area:	*Smart building management and energy-efficient HVAC optimization through real-time occupancy detection methods.*
Name of your method:	*Multimodal Occupancy Detection and HVAC Optimization*
Name and reference of original method:	***YOLOv4 for Object Detection*** *Bochkovskiy, A., Wang, C. Y., & Liao, H. Y. M. (2020). YOLOv4: Optimal Speed and Accuracy of Object Detection. arXiv preprint**arXiv:2004.10934*. *Retrieved from*https://arxiv.org/abs/2004.10934 ***Sensor Data in Occupancy Detection*** *Dong, B., & Andrews, B. (2009). Sensor-based occupancy behavioral pattern recognition for energy and comfort management in intelligent buildings. Proceedings of Building Simulation 2009, 1444–1451.*
Resource availability:	*Temperature Sensor: DHT11* *Gas Sensor: MS1100* *Arduino: Arduino Mega 2560 REV3 [A000067]* *Camera: Logitech C270 Hd Webcam*

## Background

Energy management optimization in educational facilities presents a significant challenge due to variable occupancy patterns and the substantial energy consumption of HVAC systems. Contemporary building management approaches, which typically rely on predetermined schedules or manual interventions, demonstrate notable inefficiencies in resource allocation and operational cost management.

This research presents a methodology for real-time occupancy detection that addresses the limitations of conventional approaches such as motion sensing and manual counting systems. These traditional methods exhibit constraints in accuracy, scalability, and environmental adaptability, necessitating the development of more sophisticated, non-invasive solutions.

The proposed framework leverages recent developments in computer vision and environmental sensing technologies. Specifically, this study implements a dual-modality system incorporating YOLOv4-based video analysis and CO₂ concentration monitoring. The hardware configuration comprises a Logitech C20 webcam for visual data acquisition and an MS1100 gas sensor module for atmospheric analysis, enabling robust occupancy detection even under suboptimal conditions.

The system's core computer vision component utilizes a YOLOv4 model trained on an extensive dataset comprising 20,000 annotated facial images. Implementation through OpenCV enables efficient real-time processing, while concurrent CO₂ measurement provides complementary occupancy validation. This multimodal architecture demonstrates superior reliability across diverse environmental conditions, particularly suited to dynamic educational environments.

Beyond immediate energy optimization, this methodology advances the broader objectives of intelligent building management systems. The framework's capacity for real-time occupancy assessment enables dynamic HVAC control optimization, with potential applications extending to various institutional contexts, including healthcare facilities and commercial environments.

This research contributes to the emerging field of AI-enhanced building management systems, presenting a scalable solution that addresses critical limitations in contemporary occupancy detection methodologies. The proposed framework represents a significant advancement in sustainable infrastructure management, offering both theoretical and practical implications for energy-efficient building operations.

This synergy improves occupancy estimation in real-world classrooms by mitigating challenges inherent to single-modality systems, such as lighting sensitivity in vision-based methods or latency in CO₂-based estimation.

## Method details

### Overview

This method outlines a real-time occupancy detection system that integrates video-based computer vision and gas sensor data to optimize HVAC efficiency. The dual-modality system leverages a YOLOv4 object detection model for visual analysis and an MS1100 gas sensor for environmental data collection. The following subsections provide a comprehensive description of the hardware setup, data collection protocols, system integration, and implementation details to facilitate replication.

### System architecture and integration

The multimodal system architecture integrates real-time video processing and gas sensor measurements through a synchronized data fusion pipeline. The camera continuously captures frames and relays them to a YOLOv4-powered face detection module, while the MS1100 gas sensor transmits CO₂ data to an Arduino Mega 2560 microcontroller at three-minute intervals. Both data streams are timestamped and processed concurrently within a Python-based software stack running on an NVIDIA RTX 3060 GPU.

A key component of the integration is a control logic module that compares face detection results with CO₂ concentrations to cross-validate occupancy status. For instance, if face detection shows zero occupancy but CO₂ levels are elevated above a baseline threshold, the system flags the reading for further observation or system recalibration. This redundancy ensures higher reliability, especially in scenarios with poor lighting, partial occlusions, or camera blind spots.

The synchronized design allows for real-time occupancy updates, which can then be directly interfaced with a smart thermostat control system for dynamic HVAC optimization. This architectural synergy between visual and environmental sensing provides both immediate responsiveness and robustness in varied classroom conditions.ss

### Hardware setup

1. Camera Module

• Device: Logitech C20 Webcam.
• Specifications: Captures video at 640 × 480 resolution and 23 frames per second (FPS).
• Placement: Mounted at a height of 2.5 m, providing a clear view of the entire classroom.
• Connection: The camera is connected to a local processing unit via USB.

2. Gas Sensor Module

• Device: MS1100 Gas Sensor.
• Functionality: Measures CO₂ concentration at 3-minute intervals.
• Placement: Installed at a central position in the classroom to ensure accurate readings of ambient air quality.
• Connection: Linked to an Arduino microcontroller for data collection and transmission.

3. Processing Unit

• Specifications: NVIDIA GeForce GTX 1080 Ti GPU-equipped desktop for real-time video processing.
• Software: Python-based environment with OpenCV libraries for video processing and YOLOv4 model integration.

### Pseudo code

1. Initialize camera module (Logitech C20)
2. Initialize gas sensor module (MS1100 via Arduino)
3. Load YOLOv4 face detection model (pre-trained and fine-tuned)
4. Start continuous video capture
5. Set gas sensor reading interval = 3 min
6. WHILE classroom session is active DO

a. *Capture video frame from camera*
b. *Timestamp the captured frame*

c. *Preprocess the frame:*
  i. *Apply noise reduction*
  ii. *Enhance facial features*

d. *Perform face detection using YOLOv4*
e. *Initialize face_count = 0*

f. *FOR each detection in output DO*
  i. *IF detection confidence > threshold THEN*

*- Increment face_count*

*END IF*

*END FOR*

### Data collection

To enable accurate and real-time occupancy detection, data was collected using two complementary modalities: video and environmental sensing. Continuous video feeds were captured during extended classroom sessions using a Logitech C20 webcam positioned to provide full room coverage. Each video frame was preprocessed to reduce noise and enhance facial feature visibility, thereby improving detection accuracy. Simultaneously, CO₂ concentration levels were recorded every three minutes using an MS1100 gas sensor strategically placed at the center of the room for consistent environmental sampling. All data streams—both visual and sensor-based—were timestamped to ensure synchronized integration during analysis. This dual-modality setup allowed for robust cross-validation of occupancy estimates, enhancing the reliability of the system's outputs for HVAC control optimization.

*1. Video Data*

• *Continuous video feeds were recorded during classroom sessions spanning multiple hours.*
• *Frames were preprocessed to reduce noise and enhance facial feature detection.*

*2. Gas Sensor Data*

• *CO₂ readings were collected every 3 min, providing complementary data to validate occupancy counts from video analysis.*

*3. Synchronization*

• *Video frames and gas sensor readings were timestamped to ensure seamless integration during analysis.*

### Model training and deployment

*1. YOLOv4 Training*

• *Dataset: The YOLOv4 model was pre-trained on the COCO dataset and fine-tuned using over 20,000 labeled human face images.*
• *Techniques: Data augmentation (rotation, scaling, and cropping) and hard negative mining were applied to improve model robustness.*
• *Evaluation: The model achieved over 98 % accuracy in detecting human faces in controlled environments.*

*2. Deployment*

• *The model was deployed using OpenCV to process live video frames in real time.*
• *Detected faces were counted, and confidence scores were recorded for each detection.*

## Method validation and results

Validation of the proposed methodology was conducted in a controlled university classroom environment measuring 10 m x 8 m, with a maximum capacity of 10 students. The classroom featured consistent lighting and controlled air circulation to minimize external environmental noise. Validation experiments covered three distinct scenarios: (1) static occupancy, where students remained seated throughout the session; (2) dynamic occupancy, where students entered and exited the room at irregular intervals; and (3) partial occlusion, where classroom furniture and other obstacles partially obscured the camera’s field of view. A total of 15 h of data was collected across 10 sessions, each lasting 90 min, with synchronized CO₂ sensor readings and video feeds ensuring seamless integration of multimodal data.

Performance was assessed using key validation metrics: detection accuracy (the proportion of correctly identified occupants compared to manual headcounts), false positive and negative rates, environmental robustness (system reliability under varying lighting and occlusion conditions), and system latency (time elapsed between data acquisition and output generation). These metrics provide a comprehensive evaluation of the methodology’s ability to deliver accurate and timely occupancy detection under real-world classroom conditions..

## Limitations

Please

1. Environmental Factors:

Performance may degrade in highly dynamic or poorly illuminated environments, necessitating further *optimization.*

*2. CO₂ Data Sensitivity:*

*Variations in CO₂ levels due to factors other than occupancy (e.g., ventilation) can introduce noise, which must be accounted for during calibration.*

## Ethics statements

The research methodology strictly adheres to ethical guidelines for data collection and usage. Although the system employs a camera for real-time occupancy detection, no human faces or personal identifiers were stored, saved, or processed beyond temporary detection for counting purposes. The methodology was designed to ensure complete privacy and anonymity of the individuals involved.

All students participating in the data collection process were fully informed about the purpose and procedures of the research. Prior to the study, explicit consent was obtained from all participants, ensuring their understanding and voluntary involvement. The research was conducted in compliance with institutional ethical standards, emphasizing transparency, privacy, and respect for participants’ rights.

## CRediT authorship contribution statement

**Koundinya Challa:** Writing – original draft, Writing – review & editing. **Issa W. AlHmoud:** Supervision. **Chandra Jaiswal:** Supervision. **Balakrishna Gokaraju:** Funding acquisition. **Raymond Tesireo:** Funding acquisition.

## Declaration of competing interest

The authors declare that they have no known competing financial interests or personal relationships that could have appeared to influence the work reported in this paper.

## Data Availability

Data will be made available on request.

## Appendix Supplementary materials

Image, application 1

## References

[1] Challa K. (2024). Optimizing HVAC efficiency via deep neural networks for real-time classroom occupancy. SoutheastCon.

[2] Zhen Z. (2020). Handbook of Smart Classrooms.

[3] Wen W. (2021). Machine Vision Based Occupant Counting Andrecognition System. ICTC.

[4] A. Bochkovskiy, et al., YOLOv4: optimal speed and accuracy of object detection, arXiv (2020).

[5] Stauffer C. (2004). Estimating tracking sources and sinks. Proc. COPS.

[6] S. Ren, et al., Faster R-CNN: Towards Real-Time Object Detection with Region Proposal Networks, IEEE TPAMI, 2017. 10.1109/TPAMI.2016.2577031 27295650

[7] Liu W. (2016). Single Shot Multibox Detector.

[8] Redmon J. (2016). You Only Look Once: Unified Real-Time Object Detection. CVPR.

[9] Do Q. (2019). People Counting System Using Single Camera With YOLOv2 Deep Learning Network. ICISA.

[10] Zhao Z. (2020). Classroom Occupancy Counting Based on Face Detection and Tracking with Deep CNN. CRCSI.

[11] Lin T. (2014). Microsoft COCO: Common Objects in Context.

[12] Predicting occupancy counts using physical and statistical modeling methodologies. M.S.Zuraimi.

