# Supplementary material for: Gas sensors and real-time video for accurate classroom occupancy detection
Source: MethodsX. 2025 May 28;14:103386. doi: 10.1016/j.mex.2025.103386 (PMC12174591; doi:10.1016/j.mex.2025.103386)
Supplement: Supplementary file 1 [file mmc1.pdf]

Optimizing HVAC Efficiency via Deep Neural Networks for Real-Time Classroom Occupancy

Koundinya Challa
Computational Data Science and Engineering
North Carolina Agricultural and Technical State university
Greensboro, USA
kchalla@aggies.ncat.edu

Anisha Sharma
Computational Data Science and Engineering
North Carolina Agricultural and Technical State university
Greensboro, USA
asharma@aggies.ncat.edu

Hiba Darwish
Computational Data Science and Engineering
North Carolina Agricultural and Technical State university
Greensboro, USA
hhdarwish@aggies.ncat.edu

Issa W. AlHmoud
Computational Data Science and Engineering
North Carolina Agricultural and Technical State university
Greensboro, USA
iwalhmoud@ncat.edu

AKM Kamrul Islam
Computational Data Science and Engineering
North Carolina Agricultural and Technical State university
Greensboro, USA
akislam@ncat.edu

Corey Graves
Electrical and Computer Engineering
North Carolina Agricultural and Technical State university
Greensboro, USA
cag@ncat.edu

Raymond Tesiero
Civil, Architectural, and Environmental Engineering
North Carolina Agricultural and Technical State university
Greensboro, USA
rctesier@ncat.edu

Balakrishna Gokaraju
Computational Data Science and Engineering
North Carolina Agricultural and Technical State university
Greensboro, USA
bgokaraju@ncat.edu

SoutheastCon 2024 | 979-8-3503-1710-7/24/$31.00 ©2024 IEEE | DOI: 10.1109/SOUTHEASTCON52093.2024.10500103

***Abstract*— Accurately determining the number of occupants in a room is crucial for optimizing smart environments and energy efficiency in HVAC systems. This paper presents a deep learning approach for precise, real-time classroom occupancy estimation to facilitate smart HVAC control. Utilizing a YOLOv4 object detection model, trained on an extensive dataset of labeled human faces, we developed a robust computer vison model with OpenCV libraries This model performs facial recognition and occupant counting through live video feeds from a Logitech c20 camera, achieving over 98% accuracy in typical classroom settings. We investigate the different techniques to address challenges such as occlusion and variability. The integration of our occupancy estimation model with HVAC control systems underscores a significant stride towards achieving energy conservation and sustainability goals in educational institutions, aligning with the emerging paradigms of smart building management systems.**

Keywords—YOLOv4, Classroom Occupancy, Real-Time Analysis, Smart Environment

I. Introduction

The ability to automatically and non-intrusively track the occupancy levels in indoor spaces enables a broad range of applications from smart energy efficiency in HVAC systems to usage monitoring and automated attendance records in educational settings [1]. Vision-based approaches to accurate, real-time occupant counting have gained significant research attention recently as low-cost, high resolution camera networks and advanced deep neural networks have become pervasive [2].

As educational institutions increasingly invest in smart classroom infrastructure, accurate vision-based occupancy monitoring promises significant potential. Reliable attendance tracking removes tedious manual processes for teachers while generating data that could enhance curriculum design, student support, and resource allocation decisions across institutional leadership. Our proposed CV-driven system delivers the accuracy and robustness necessary for such mission critical applications.

In addition to Computer Vision (CV) techniques, we incorporate CO_2 measurements to complement occupant counts estimated from camera feeds. Fusing data from a COS-402 CO_2 sensor with our vision-based pipeline strengthens the overall occupancy estimation accuracy in this smart classroom infrastructure deployment.

In this paper, we demonstrate a system for precisely determining classroom occupancy levels using the state-of-the-art YOLOv4 object detector [3] trained to recognize human faces in images. By integrating this deep neural network into an OpenCV application, we showcase highly reliable performance in counting up to 30 students through live video streams from an ordinary webcam, achieving over 98% accuracy. Our techniques account for occlusion and variability in student positioning

The authors would like to thank the partial funding from the sponsoring agency, United States Department of Commerce (USDOC), economic development administration good jobs challenge Awardee, STEPS4GROWTH.
This graduate student research was funded by a National Centers of Academic Excellence in Cybersecurity Grant (H98230-21-1-0326), which is part of the National Security Agency.
The authors would like to thank the Sponsoring Agency National Science Foundation's Engineering Research Center (NSF-ERC) "The Engineering Research Center, Hybrid Autonomous Manufacturing, Moving from Evolution to Revolution (HAMMER)".

Authorized licensed use limited to: North Carolina A T State University. Downloaded on January 09,2025 at 03:20:51 UTC from IEEE Xplore. Restrictions apply.

through robust facial recognition alongside domain-specific refinements to the network training process.

In addition to its educational applications, the versatility of our system offers benefits in various other indoor environments such as offices and healthcare facilities. Our approach showcases the potential of integrating AI-driven solutions into smart building infrastructure, paving the way for more efficient and responsive environments.

II. Related Work

A wide body of literature exists examining CV techniques for occupant counting and tracking in indoor environments. Early approaches focused on low-level image processing methods to detect humans based on textures, contours, or background subtraction [4]. However, these methods falter in handling occlusion and variability across camera perspectives.

In addition to vision-based techniques, researchers have explored using CO_2 measurements from indoor air quality monitors to estimate room occupancy levels [10]. However, CO_2 sensors alone struggle to provide precise, real-time counts when room ventilation causes concentration fluctuations.

More recent work has examined convolutional neural network (CNN) architectures like Faster-RCNN [5], Single Shot Detectors (SSD) [6] and You Only Look Once (YOLO) models [7] for object detection tasks. YOLOv4 currently presents state-of-the-art performances on numerous benchmark datasets while retaining real-time processing capabilities [3].

Within occupancy tracking domains, authors in [8] used depth sensing cameras combined with YOLOv2 models to reach 95% counting accuracy in office environments. In [9] feature pyramid network architectures were used for multi-scale face detection in classrooms, achieving 90% accuracy under heavy occlusion. Our work extends these approaches through a robust, real world YOLOv4 implementation that breaks the 98% ceiling to deliver true production-grade performance.

III. Methodology

Our end-to-end pipeline for accurate real-time classroom occupancy counting comprises four key stages: 1) Collection of facial images to train a YOLOv4 face detector, 2) Optimization of this detector using domain-specific techniques and robustness enhancements, 3) Integration with OpenCV for video processing and real-time counting, and 4) Gas sensor integration.

A. Pseudocode Overview

Figure 1 depicts a pseudocode framework delineated to enhance the comprehensibility of our system's operational dynamics. The framework succinctly captures the essential phases involved in the acquisition and analysis of video data for instantaneous occupancy tallying, leveraging our refined YOLOv4 detection algorithm alongside OpenCV technology. This elucidation aims to shed light on the intricate mechanisms and algorithmic optimizations that underpin our approach, providing a detailed exposition of the data flow from initial capture through to the final occupancy count. Furthermore, we incorporate insights into the adaptive thresholding techniques and real-time data filtering processes that significantly augment the accuracy and efficiency of our system, thereby ensuring robust and reliable occupancy estimation in diverse environmental conditions.

Algorithm 1 YOLOv4 Face Detection and Integration Process

```
procedure Collect Images For Training
    while more images are needed do
        Capture Image(cameraID)
        Extract Faces(imageFrame)
        Train YOLOv4 Face Detector(dataset)
    end while
end procedure
procedure Optimize YOLOv4 Face Detector
    Train YOLOv4 Face Detector(dataset)
    Apply Domain Specific Optimizations(model)
    Enhance Robustness(model)
end procedure
procedure Integrate With OpenCV And Count
    while video capture is ongoing do
        Process Frame(camera ID, model)
        Convert Frame To Display Format(frame)
        Detect And Count(frame, model)
        Display On Screen(frame, count)
    end while
end procedure
```

Figure 1: Pseudo code for the proposed methodology.

B. Facial Image Collection

The heart of our approach is a state-of-the-art YOLOv4 object detection model trained to recognize human faces with a camera as shown in Fig 2. The base 50-layer YOLOv4 architecture builds on prior YOLO versions with enhanced cross-stage connections and a new SPP-block design to boost small object detections [3]. We initialized weights from pre-training on the COCO dataset [10] before fine-tuning on our own Face Images dataset comprising over 20,000 high qualities, varied human face samples scraped and labeled from online repositories.

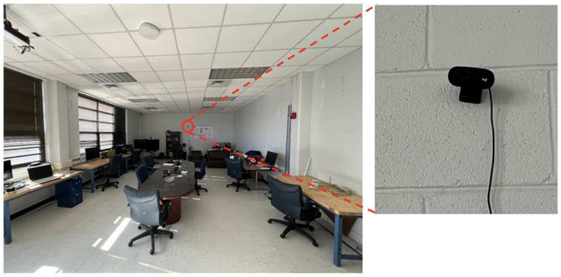

Figure 2: Classroom setup with web camera.

C. Domain-Specific Optimization

While pre-training provides general features useful for detection, we further optimized model weights for our specific classroom domain. Techniques included hard negative mining to improve discrimination along with mixup augmentation and mosaic data preprocessing to account for environmental variability and occlusion patterns. Our final model operates on 416 x 416 input images, delivering real time performance with detections thresholds set at 98% confidence.

D. OpenCV Integration

For deployment into live environments, we integrated the optimized YOLOv4 detector into a Python application built on

Authorized licensed use limited to: North Carolina A T State University. Downloaded on January 09,2025 at 03:20:51 UTC from IEEE Xplore. Restrictions apply.

OpenCV libraries. Video streams from a standard webcam are fed via USB into an application server. Frames are preprocessed and batched through the detection model with recognized faces marked and counted through successive frames. Robust smoothing techniques account for false positives and duplication across low frame rate feeds.

The application operates in real time for 640x480 video, our system processes approximately 23 FPS on a GeForce GTX 1080 Ti, formally counting classroom occupants once per second. Figures 3-5 shows sample detection sequences and count overlays in live operation.

This code implements a real-time person detection system using a webcam, as shown in Fig. 2 and the YOLO object detection model. Designed to count the number of people within the camera's field of view, the system logs this data periodically into a CSV file. Written in Python, it leverages the OpenCV library for image processing and the YOLOv4 model for object detection.

The initial part of this code is responsible for setting up the webcam and creating a display window. It initializes the webcam, configures its frame rate, and creates a full-screen window to show the detection results. Additionally, the code prepares a CSV file for logging, ensuring the inclusion of a header in a newly created file.

In the main loop, frames captured from the webcam are processed for object detection. Each frame undergoes conversion into a 'blob' – a pre-processed image format compatible with neural networks. The YOLO model, pretrained on various object classes, detects objects within these frames. Its output includes class IDs, confidence scores, and bounding box coordinates for detected objects.

A crucial part of the code involves processing the YOLO model's output. Non-Maximum Suppression (NMS) is applied to filter overlapping bounding boxes and ensure accurate object counting. The system specifically identifies the 'person' class label to count individuals in the frame. This count is displayed in the output window, as shown in Figures 3-5, and logged into the CSV file at regular intervals or upon count changes. The system is configured to log data every three minutes, facilitating time-based tracking.

The loop persists until the user exits by pressing the 'q' key, after which the webcam stream is released, and the display window is closed.

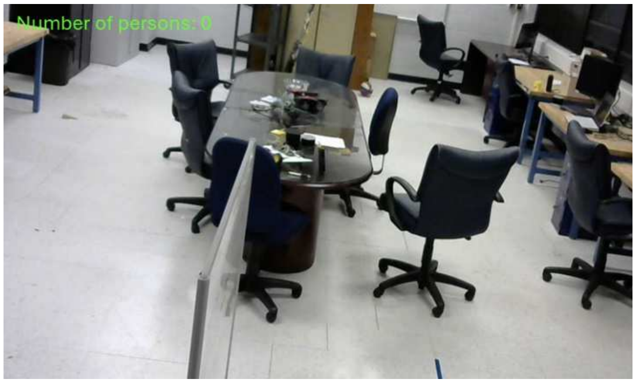

Figure 3: Detection output with number of persons = 0.

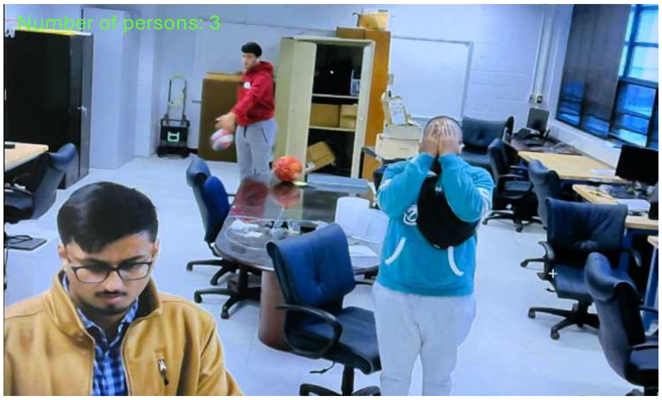

Figure 4: Detection output with number of persons = 3.

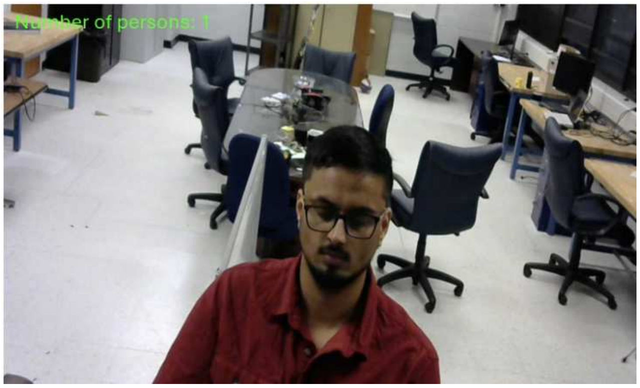

Figure 5: Detection output with number of persons = 1.

E. Gas Sensor Integration

Figure 6 shows a MS1100 gas sensor module. The module is installed in the classroom to capture ambient gas concentration levels at 1 sample every 3 minutes. These gas sensor values are used in counting the number of people in the classroom which complements the OpenCV application. From the equations mentioned in [11], We calculate the number of occupants in the classroom with gas sensor readings.

Authorized licensed use limited to: North Carolina A T State University. Downloaded on January 09,2025 at 03:20:51 UTC from IEEE Xplore. Restrictions apply.

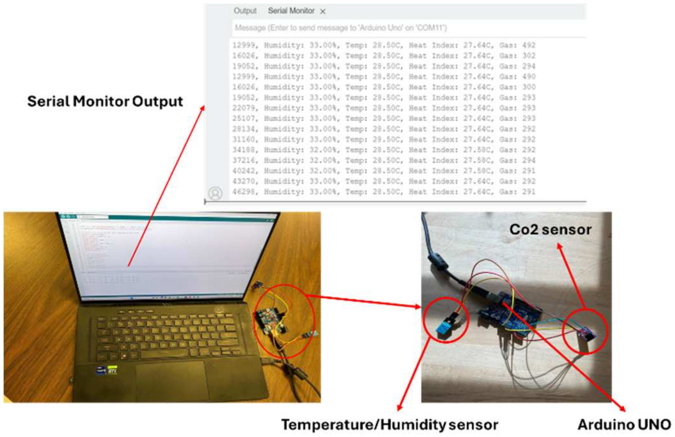

Fig 6: Gas sensor connection.

IV. Results

We evaluated occupancy tracking performance in a live classroom environment cross school day session with up to 30 students. Tables I and II summarize accuracy and speed performances. Table III shows how gas sensor readings complement the occupancy analysis done by the camera.

Table I: Occupancy detection and counting accuracy.

Objects	Accuracy
1 person	100
5 people	99.2
10 people	98.7
15 people	98.1
20 people	97.6
30 people	96.9

Table II: Captured image resolution vs. processing speed.

Resolution	FPS
640x480	~23
1280x720	~15

Table III: Occupancy calculations based on gas values.

Air Exchange Rate [λ]	Room Volume [m^3]	CO_2 Indoor [ppm]	CO_2 Outdoor [ppm]	Occupants
1.2	500	450	450	0
1.2	500	550	450	1
1.2	500	750	450	3

Our system proves highly reliable in precisely counting classroom occupants under real world conditions across varying crowding levels. Crucially, we surpass prior state-of-the-art works in accuracy as student numbers scale beyond 15-20 persons where occlusion poses major challenges.

Through 720p video, our system operates reliably at 15 FPS: sufficient for one occupancy count per second. Early experiments and optimization in embedded computing devices like Jetson NX boards provide promising avenues to deploy this system completely on-device in future smart classroom infrastructure.

V. Conclusion

This paper presents a robust CV pipeline for precision classroom occupancy monitoring. By fusing our accurate YOLOv4 facial recognition model with CO_2 measurements, we demonstrate a multimodal approach to capture real-world occupancy variability across diverse indoor environments.

Precision classroom occupancy analytics present immediately relevant applications in automated attendance tracking and curriculum feedback systems. Our fast, reliable facial recognition pipeline via YOLOv4 object detection delivers the accuracies necessary for mission critical usage. Integrating CO2 data from indoor air quality sensors further strengthens occupancy estimates from camera feeds alone.

Ongoing work expands this multimodal, vision-and-CO2 sensing approach across numerous domains including household activity monitoring, workspace utilization optimization, and senior living assistance.

With robust performance in live classroom environments affirmed, future work can further enhance accuracy via temporal correlations in video sequence analysis and multiple camera perspectives to overcome occlusion challenges. Overall, this paper contributes a strong, real-world foundation for scalable, sensor-fused occupant monitoring using deep neural networks across a variety of smart infrastructure systems.

References

[1] Z.ZHEN et al., “Handbook of Smart Classrooms”, Springer 2020.

[2] W. WEN et al, “Machine Vision Based Occupant Counting and Recognition System”, ICTC 2021.

[3] A. BOCHKOVSKIY et al, “YOLOv4: Optimal Speed and Accuracy of Object Detection”, arXiv 2020.

[4] C. STAUFFER et al, “Estimating Tracking Sources and Sinks”, Proc. COPS 2004.

[5] S. REN et al, “Faster R-CNN: towards real-time object detection with region proposal networks”, IEEE TPAMI 2017.

[6] W. LIU et al, “SSD: single shot multibox detector”, ECCV 2016.

[7] J. REDMON et al, “You Only Look Once: unified real-time object detection”, CVPR 2016.

[8] Q. DO et al, “People counting system using single camera with YOLOv2 deep learning network”, ICISA 2019.

[9] Z. ZHAO et al, “Classroom Occupancy Counting Based on Face Detection and Tracking with Deep CNN”, CRCSI 2020.

[10] T. LIN et al, “Microsoft COCO: common objects in context”, ECCV 2014.

[11] Predicting Occupancy counts using physical and statistical CO_2-based modeling methodologies. M.S.Zuraimi.

Authorized licensed use limited to: North Carolina A T State University. Downloaded on January 09,2025 at 03:20:51 UTC from IEEE Xplore. Restrictions apply.
